# Usefulness of Low-Dose Splenic Irradiation prior to Reduced-Intensity Conditioning Regimen for Hematopoietic Stem Cell Transplantation in Elderly Patients with Myelofibrosis

**DOI:** 10.1155/2016/8751329

**Published:** 2016-10-20

**Authors:** Etsuko Matsubara, Jun Yamanouchi, Riko Kitazawa, Taichi Azuma, Hiroshi Fujiwara, Takaaki Hato, Masaki Yasukawa

**Affiliations:** ^1^Department of Hematology, Clinical Immunology and Infectious Diseases, Ehime University Graduate School of Medicine, Toon, Japan; ^2^Department of Molecular Pathology, Ehime University Graduate School of Medicine, Toon, Japan

## Abstract

The Janus kinase (JAK) 1 and 2 inhibitor, ruxolitinib, was recently approved in Japan and has been effective in many patients with myelofibrosis (MF). Although the inhibitor decreases splenomegaly and relieves MF-related symptoms, allogeneic hematopoietic cell transplantation (HCT) remains as the only curative therapy for MF. The presence of splenomegaly has been reported as a risk factor for graft failure, delayed engraftment, and poor survival. Here, we report two elderly MF patients with massive splenomegaly and a JAK2 V617F mutation. These patients underwent splenic irradiation to decrease splenomegaly prior to HCT with a reduced-intensity conditioning (RIC) regimen. Massive splenomegaly gradually decreased by 4 Gy splenic irradiation. The subsequent RIC regimen involved 4 Gy total body irradiation and fludarabine and intravenous busulfan. In both patients, engraftment failure did not occur, and complete remission was achieved. The splenomegaly decreased, and MF-related symptoms were resolved. Furthermore, the JAK2 V617F mutation disappeared, and fibrosis in the bone marrow regressed. We suggest that splenic irradiation prior to the RIC regimen for HCT in elderly MF patients with massive splenomegaly is safe. Furthermore, the HCT protocols with splenic irradiation should be considered for patients who have not shown clinical benefits to optimal medical management such as treatment with ruxolitinib.

## 1. Introduction

Myelofibrosis (MF) is a clonal myeloproliferative disorder that is characterized by bone marrow fibrosis, extramedullary hematopoiesis, progressive anemia, excessive proliferation of megakaryocytes and granulocytes, and splenomegaly. MF can develop from* de novo* primary myelofibrosis (PMF), polycythemia vera (post-PV MF), or essential thrombocythemia (post-ET MF). The median overall survival (OS) estimates for the risk categories from the Dynamic International Prognostic Scoring System (DIPSS) are 14.2 years, 4 years, and 1.5 years for the intermediate-1, intermediate-2, and high-risk groups, respectively. The median OS estimate for the low-risk group is unavailable. Conventional therapies, such as chemotherapy, administration of erythropoietin and/or androgens, splenic irradiation, and splenectomy, do not prolong OS.

The Janus kinase (JAK) 2 V617F mutation was identified as the most common molecular abnormality in MF patients in 2005 [1–3]. The gain-of-function JAK2 V617F mutation is present in approximately 50% of patients with PMF. In phase III controlled trials, ruxolitinib, a selective inhibitor of JAK1/2, provided significant clinical benefits in patients with MF by reducing spleen size and improving MF-related symptoms and OS [4–6]. Ruxolitinib was approved by the US Food and Drug Administration for the treatment of intermediate- and high-risk MF in November 2011 and subsequently approved in Japan in 2014. However, allogeneic hematopoietic cell transplantation (HCT) is the only curative therapy for MF since ruxolitinib does not eliminate the myeloproliferative neoplasm (MPN) clone, reduce JAK2 V617F allele burden, or regress bone marrow fibrosis in most patients. Since myeloablative conditioning regimens cannot be used for elderly patients, the use of reduced-intensity conditioning (RIC) regimens is suggested instead [7]. A cohort analysis from the Center for International Blood and Marrow Transplant Research (CIBMTR) evaluated the outcomes and prognostic factors in 233 primary MF patients undergoing HCT with RIC regimens. The probability of survival for these patients at 5 years was 47%. In multivariate analysis, donor type was a factor associated with survival. In DIPSS intermediate-2 and high-risk patients, nonrelapse mortality was higher and survival was inferior compared to DIPSS low/intermediate-1 risk patients. These findings indicate that HCT with RIC regimens is a potentially curative option for patients with MF and that donor type is the most important factor influencing survival in these patients [8]. In addition, the presence of massive splenomegaly is a risk factor for graft failure, delayed engraftment, and poor survival [9]. Although several strategies were developed for reducing spleen size prior to HCT, their efficacy and safety have not been established due to the small number of cases [10, 11]. Here, we report two elderly MF patients with massive splenomegaly and JAK2 V617F mutation who successfully underwent splenic irradiation prior to allogeneic HCT with the RIC regimen.

## 2. Case Reports

**Figure 1:**
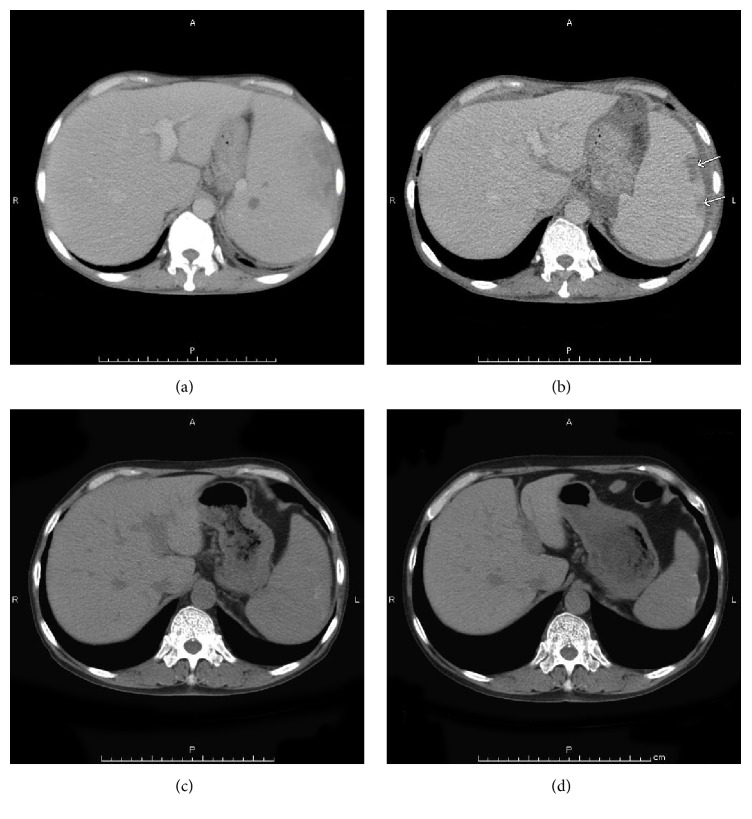
*Case  1.* Computed tomography (CT). (a) Before HCT, the CT showed severe splenomegaly. (b, c, d) After HCT, the massive splenomegaly was gradually reduced. (b) Three months. (c) Six months. (d) Twelve months. The arrow shows infarction.

**Figure 2:**
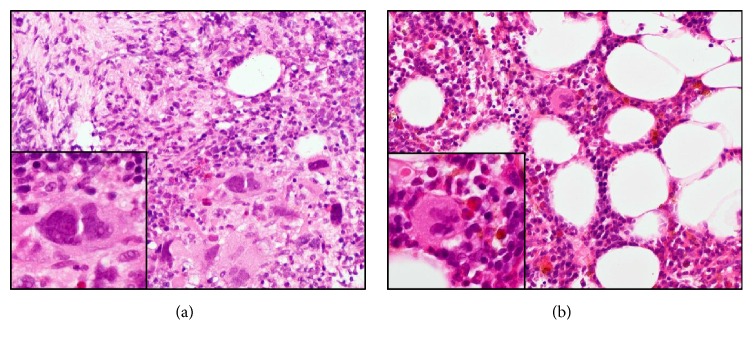
*Case  1.* Histology of bone marrow (HE stain). (a) Before HCT, the bone marrow biopsy showed severe fibrosis (MF-2), and the megakaryocytes had changed morphologically. (b) After HCT, the marrow specimen showed that the fibrosis regressed (MF-0), and the morphological abnormalities in megakaryocytes had disappeared.

*Case  1.* Ten years before, a 67-year-old man was diagnosed with PMF. Although he had been treated with hydroxyurea, his spleen had progressively enlarged and extended to his pelvis (Figure 1(a)). He exhibited fatigue, abdominal pain, and transfusion-dependent anemia. Laboratory blood tests showed bicytopenia (WBC, 6.6 × 10^3^/L; Hb, 7.6 g/dL; platelets, 7.4 × 10^10^/L) and leukoerythroblastosis. A bone marrow biopsy showed hypocellular marrow with severe fibrosis and changes in megakaryocyte morphology (Figure 2(a)). We detected the JAK2 V617F mutation in the peripheral blood using allele-specific PCR. The disease was classified as “intermediate-2 risk,” according to the prognostic classification of the DIPSS, and the patient was evaluated as a candidate for HCT. After written informed consent was obtained, he was treated with 4 Gy splenic irradiation on day −7 to day −6 prior to HCT. With splenic irradiation, there was a rapid reduction in splenomegaly, and the spleen shrank to 10 cm below the left costal margin. Furthermore, the MF-related abdominal pain was alleviated. The conditioning regimen consisted of fludarabine (Flu) 30 mg/m^2^ × 5 (day −6 to day −2), intravenous busulfan (Bu) 3.2 mg/m^2^ × 2 (day −3 to day −2), and 4 Gy of total body irradiation (TBI) on day −5 to day −4. The patient underwent HCT from a human leukocyte antigen- (HLA-) mismatched unrelated donor (mismatch in HLA-DRB1). The infused bone marrow contained 3.7 × 10^8^/kg nucleated cells. Graft-versus-host disease (GVHD) prophylaxis consisted of continuous infusion of tacrolimus and short-term methotrexate. Neutrophil engraftment occurred on day 21. The chimerisms in the bone marrow and peripheral blood were 100% donor. A bone marrow aspiration and biopsy showed complete remission. A skin biopsy assessment showed that Grade I acute GVHD of the skin occurred on day 34. There was no GI toxicity as a result of the splenic irradiation. Furthermore, the JAK2 V617F mutation was not detected after the neutrophil engraftment. A bone marrow biopsy specimen showed that the fibrosis regressed on day 377 (Figure 2(b)). Although chronic GVHD of the liver and mouth occurred 20 months after transplantation, the symptoms disappeared following the administration of corticosteroid. The patient's spleen gradually decreased in size until it was not palpable, and computed tomography showed that the massive splenomegaly was reduced (Figures 1(b), 1(c), and 1(d)). The patient is still alive without relapse for 52 months after HCT.

**Figure 3:**
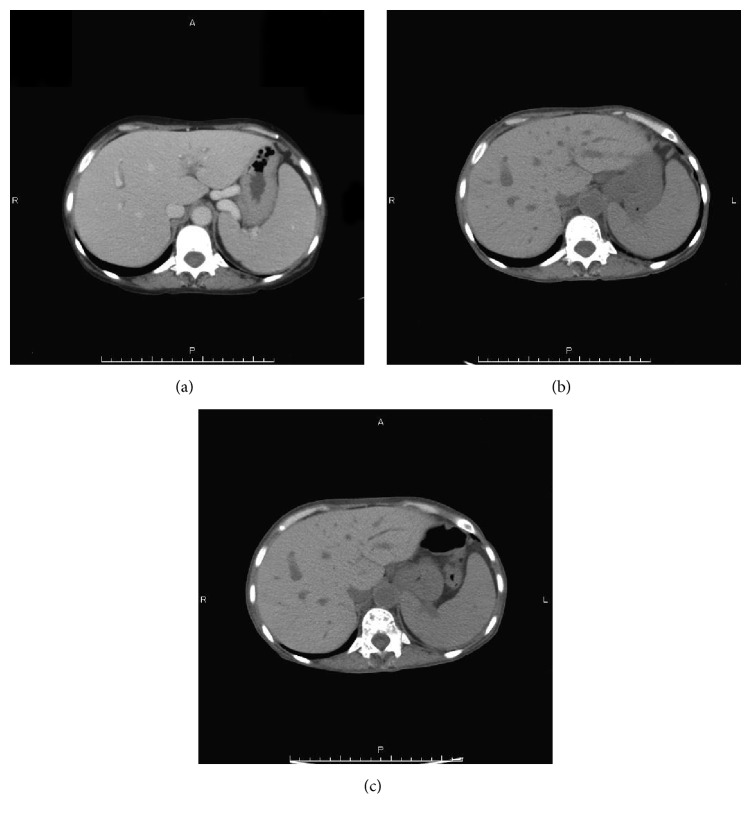
*Case  2.* CT. (a) Before HCT, the CT showed severe splenomegaly. (b, c) After HCT, the massive splenomegaly was reduced. (b) Three months. (c) Six months.

**Figure 4:**
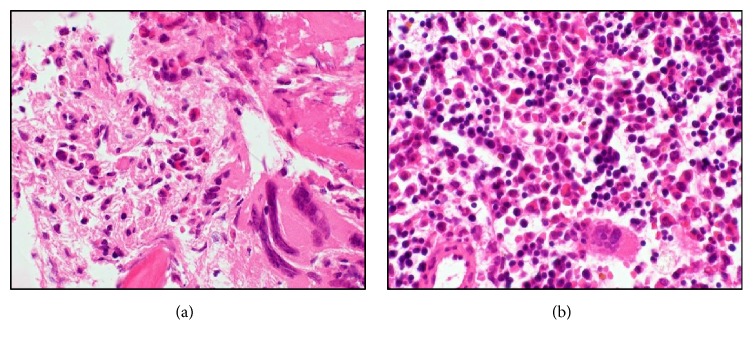
*Case  2.* Histology of bone marrow (HE stain). (a) Before HCT, the bone marrow biopsy showed secondary MF. (b) After HCT, the marrow specimen showed that the fibrosis regressed, and the morphological abnormalities in megakaryocytes had disappeared.

*Case  2.* A 61-year-old woman with progressive leukocytosis and splenomegaly (Figure 3(a)) was diagnosed with ET 20 years ago. Additionally, she was diagnosed with post-ET MF 5 years ago. Although she was treated with hydroxyurea, her spleen had progressively enlarged and extended 10 cm below the left costal margin. She exhibited MF-related symptoms such as fatigue, abdominal pain, and transfusion-dependent anemia. Laboratory blood tests showed pancytopenia (WBC, 3.0 × 10^3^/L; Hb, 7.0 g/dL; platelets, 7.1 × 10^10^/L) and leukoerythroblastosis. A bone marrow biopsy showed hypocellular marrow with severe fibrosis (Figure 4(a)). We detected the JAK2 V617F mutation in the peripheral blood. The disease was classified as “intermediate-2 risk” according to the prognostic classification of the DIPSS, and she was evaluated as a candidate for HCT. After written informed consent was obtained, she was treated with 4 Gy splenic irradiation on day −9 to day −8 prior to HCT. During splenic irradiation, her splenomegaly decreased, and the abdominal pain gradually improved. The conditioning regimen consisted of Flu 30 mg/m^2^ × 5 (day −6 to day −2), Bu 3.2 mg/m^2^ × 2 (day −6 to day −5), and 4 Gy of TBI on day −6 to day −5. The patient underwent HCT from an HLA-matched unrelated donor. The infused bone marrow contained 2.3 × 10^8^/kg nucleated cells. Neutrophil engraftment occurred on day 30. Grade III acute GVHD of the gut occurred on day 76 along with chronic GVHD of the liver. There was no GI toxicity as a result of the splenic irradiation. The chimerisms in the bone marrow and peripheral blood were 100% donor. A bone marrow aspiration and biopsy showed complete remission, and fibrosis regressed on day 340 (Figure 4(b)). The JAK2 V617F mutation was not detected since the neutrophil engraftment. The patient's spleen gradually decreased in size until it was not palpable, and computed tomography showed that the splenomegaly was reduced (Figures 3(b) and 3(c)).

## 3. Discussion

The predictive OS factors for patients with MF who undergo HCT are age, performance status, and severity of splenomegaly [12]. In a study that consisted of 46 patients with PMF who underwent HCT with the RIC regimen, the factors that decreased survival were frequent blood cell transfusions, massive spleen size, alternative donors, and high-risk disease state [13]. Since spleen size is one of the factors affecting survival rate, methods to reduce its size prior to HCT are being developed.

Although splenectomy is effective for MPN-related splenic pain, it is associated with operative mortality [14]. In one study, splenomegaly was associated with delayed engraftment, but splenectomy prior to HCT promoted faster leukocyte engraftment and had no impact on survival [15]. However, there is another report that suggests patients who had undergone splenectomy before HCT had a lower post-HCT mortality after adjustment for the DIPSS risk (hazard ratio 0.57, *P* = .15) [16].

Splenic irradiation is an attractive treatment since it decreases splenomegaly and relieves MF-related symptoms in more than 90% of patients. However, the median duration of the response is only 6 months, and treatment-related cytopenia, which can be lethal, was observed in approximately 40% of high-dose irradiated patients. Since ruxolitinib was not yet approved in Japan at the time of the study and a safe method to use it before HCT was not yet known, we decided to use splenic irradiation prior to HCT to reduce massive splenomegaly. Furthermore, we decided on the timing and dose of the splenic irradiation prior to HCT by referring to a study that reported low-dose induction splenic radiotherapy was effective in accelerated-phase MF [17]. We chose the type of RIC regimen by referring to the data by the CIBMTR that showed conditioning regimens were FluBu-based in 38% and FluTBI-based in 22% of patients [8].

In addition to the successful donor cell engraftments that occurred without severe complications, we showed that hematological and molecular remissions were achieved in MF patients who had splenic irradiation prior to HCT with the RIC regimen. Furthermore the fibrosis in the bone marrow regressed after HCT. Our results were similar to Kröger et al., who reported that rapid regression of bone marrow fibrosis was associated with donor cell chimerism, fewer relapses, and favorable survival, which were independent of the DIPSS risk score at transplantation [18].

So far, no conclusions have been reached concerning the use of ruxolitinib in the peritransplant period due to the unpredictable and severe responses from patients, rebound of MF symptoms and cytokine storm reactions resulting from its discontinuation, and lack of long-term response data. The first reported study of ruxolitinib administration prior to HCT included 14 patients with MF [10]. During treatment with ruxolitinib, MF-related symptoms were decreased in 71.4%, the palpable spleen was reduced in 64%, and engraftment occurred in 93% of the patients. OS, event-free survival, and treatment-related mortality were 78.6%, 64%, and 7%, respectively, at the 9-month follow-up. Anemia and thrombocytopenia caused by ruxolitinib were observed within a few months after initiating therapy. While managing cytopenia, the disease state of accelerated-phase MF worsened [10]. In another study, Stübig et al. reported that survival improved for patients who responded to ruxolitinib [11]. However, Robin et al. found that 7 of 22 patients developed serious adverse events (e.g., cardiogenic shock and tumor lysis syndromes), and 2 patients died due to severe GVHD. Although this study was closed, the trial design has been modified and is currently in recruitment (NCT01795677) [19]. Recently, there has been a large retrospective study of patients who received JAK1/2 inhibitors prior to HCT. The OS for these patients at 2 years was 61%. Furthermore, the OS was 91% for those who experienced clinical improvement with JAK1/2 inhibitors and 32% for those who developed leukemic transformation on JAK1/2 inhibitors. In multivariate analysis, response to JAK1/2 inhibitors, DIPSS, and donor type were significant predictors of survival. Adverse events were more common in patients who started tapering or ended their regular dose for more than 6 days before conditioning therapy [20]. Whether JAK1/2 inhibitors should be continually administered close to the start of conditioning therapy is being investigated in the ongoing trials by the Myeloproliferative Disorders Research Consortium (NCT01790295) and the Fred Hutchinson Cancer Research Center (NCT02251821).

In both our cases, the JAK2 V617F mutation disappeared after engraftment. The JAK2 V617F mutation can play an important role as a marker for residual or recurrent disease after HCT. Measuring the JAK2 p.V617F allele burden soon after transplantation may be an important predictive parameter for survival since patients with a JAK2 p.V617F allele burden of >1% have a significantly higher risk of relapse and a poorer OS [21].

Our report is the first to suggest that low-dose splenic irradiation prior to HCT with the RIC regimen in elderly patients is not only safe, but also effective for engraftment and deep remission of MF. However, due to the small number of cases, it has not been clarified whether splenic reduction by irradiation can reduce time to engraftment or decrease graft failure. Further studies are required to evaluate these strategies.
